# Supplementary material for: Identification of photoperiod-induced specific miRNAs in the adrenal glands of Sunite sheep (Ovis aries)
Source: Front Vet Sci. 2022 Jul 22;9:888207. doi: 10.3389/fvets.2022.888207 (PMC9354845; doi:10.3389/fvets.2022.888207)
Supplement: Supplementary file 1 [file Data_Sheet_1.ZIP › Supplementary file/miRDeep2 file.zip!/miRDeep2 file/Know_Structure/SPLP42A2/oar-mir-136.pdf]

miRBase precursor : oar-mir-136
Total read count : 8891
oar-miR-136 read count : 2483
remaining reads : 6408

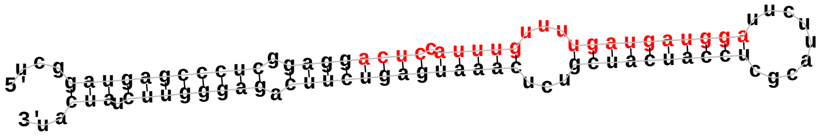

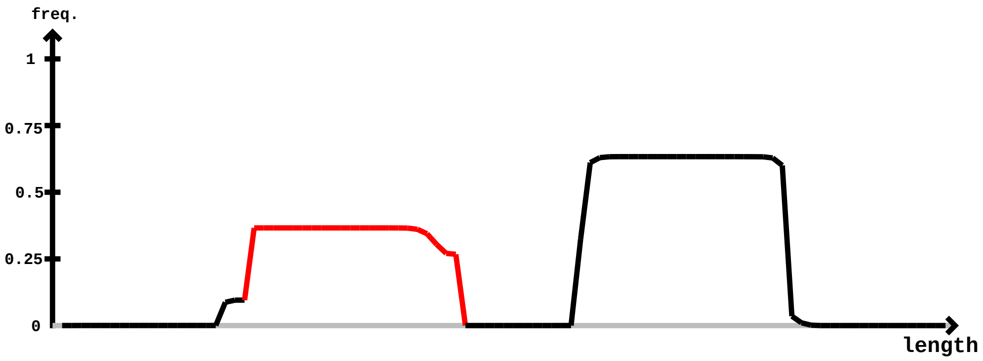

oar-miR-136

5'- ucggaugagcccucggaggacuccauuuguuuugaugauggauucuuacgcuccaucaucgucucaaaugagucuucagaggguucuaucau -3'	exp		
...(((((((((((.((((((((.(((((...((((((((((.........))))))))))...))))))))))))).)))))))).))))..	reads	mm	sample
ucggaugagcccucggagg...	1	0	seq
.cggaugagcccucggagg...	2	0	seq
................aggacuccauuuguuuug..	2	0	seq
................aggacuccauuuguuuuga...	4	0	seq
................aggacuccauuuguuuugau..	41	0	seq
................aggacuccauuuguuuugaug...	145	0	seq
................aggacuccauuuguuuugauga..	337	0	seq
................aggacuccauuuguuuugaugau...	241	0	seq
................aggacuccauuuguuuugaugaug..	3	0	seq
.................ggacuccauuuguuuugau..	1	0	seq
.................ggacuccauuuguuuugaug...	2	0	seq
.................ggacuccauuuguuuugauga..	18	0	seq
.................ggacuccauuuguuuugaugau...	50	0	seq
.................ggacuccauuuguuuugaugaug..	1	0	seq
...................acuccauuuguuuugaugau...	9	0	seq
...................acuccauuuguuuugaugaug..	25	0	seq
...................acuccauuuguuuugaugaugg...	2375	0	seq
...................acuccauuuguuuugaugaugga..	1	0	seq
....................cuccauuuguuuugaugaug..	1	0	seq
....uccaucaucgucucaaaug......................	1	0	seq
....caucaucgucucaaaug......................	1	0	seq
....caucaucgucucaaauga.....................	1	0	seq
....caucaucgucucaaaugag....................	1	0	seq
....caucaucgucucaaaugagu...................	29	0	seq
....caucaucgucucaaaugaguc..................	197	0	seq
....caucaucgucucaaaugagucu.................	2635	0	seq
....caucaucgucucaaaugagucuu................	22	0	seq
....aucaucgucucaaaugag....................	2	0	seq
....aucaucgucucaaaugagC...................	3	1	seq
....aucaucgucucaaaugagu...................	4	0	seq
....aucaucgucucaaaugaguc..................	53	0	seq
....aucaucgucucaaaugagucu.................	2350	0	seq
....aucaucgucucaaaugagucuu................	129	0	seq
....aucaucgucucaaaugagucuuc...............	4	0	seq
....ucaucgucucaaaugagC...................	1	1	seq

oar-miR-136

```
ucggaugagcccucggaggacuccauuuguuuugaugauggauucuuacgcuccaucaucgucucaaaugagucuucagaggguucuaucau
.......................................................ucaucgucucaaaugagucu.................    37    0    seq
.......................................................ucauUgucucaaaugagucu.................    1     1    seq
.......................................................ucaucgucucaaaugagucuu................    69    0    seq
.......................................................ucaucgucucaaaugagucuuc...............    55    0    seq
.......................................................ucaucgucucaaaugagucuuca..............    4     0    seq
........................................................caucgucucaaaugagucuu................    2     0    seq
........................................................caucgucucaaaugagucuuc...............    19    0    seq
........................................................caucgucucaaaugagucuuca..............    9     0    seq
.........................................................aucgucucaaaugagucu.................    2     0    seq
.........................................................aucgucucaaaugagucuu................    1     0    seq
```
